# Supplementary material for: Inhibition Mechanism of L-Cysteine on Maillard Reaction by Trapping 5-Hydroxymethylfurfural
Source: Foods. 2021 Jun 16;10(6):1391. doi: 10.3390/foods10061391 (PMC8234683; doi:10.3390/foods10061391)
Supplement: Supplementary file 1 [file foods-10-01391-s001.zip › foods-1218996-SI.pdf]

Supplementary materials

Table S1. Gradient Elution Procedure for HPLC-DAD

Time (min)	Flow rate (mL/min)	Mobile Phase A (%)	Mobile Phase B(%)
0	0.6	97 %	3 %
7.5	0.6	97 %	3 %
7.6	0.9	97 %	3 %
16.0	0.9	97 %	3 %
16.1	1.2	97 %	3 %
22.0	1.2	97 %	3 %
22.5	0.6	97 %	3 %
25.0	0.6	97 %	3 %

Table S2. Gradient Elution Procedure for UPLC System

Time (min)	Phase A (%)	Phase B (%)	Flow Rate (mL/min)
0.00	90.0	10.0	0.300
2.00	80.0	20.0	0.300
4.00	70.0	30.0	0.300
6.00	60.0	40.0	0.300
8.00	35.0	65.0	0.300
8.10	90.0	10.0	0.300
11.00	90.0	90.0	0.300

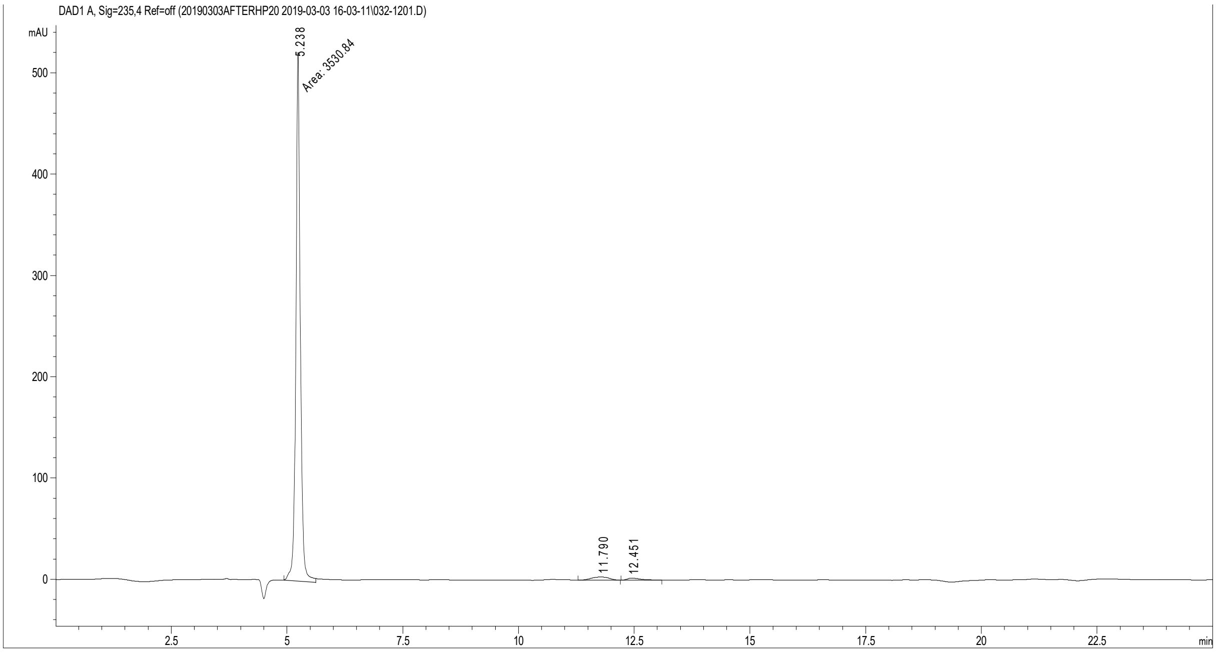

Figure S1. Chromatogram of purified DTH

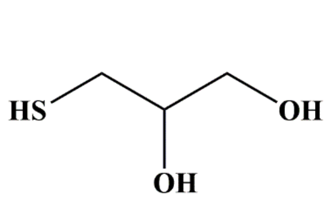

1-mercaptoglycerol

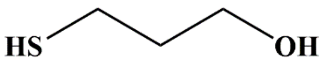

3-mercapto-1-propanol

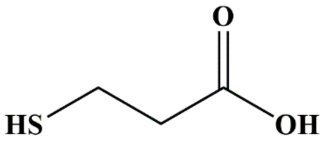

3-mercaptopropanoic acid

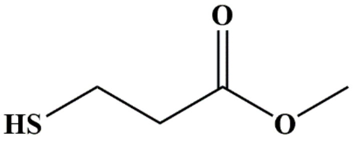

3-mercaptopropionic acid methyl ester

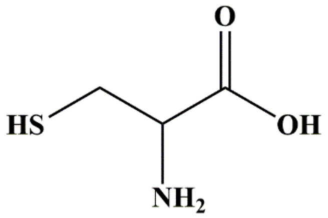

(2R)-2-amino-3-mercapto propanoic acid

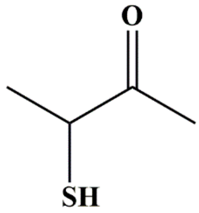

3-mercapto-2-butanone

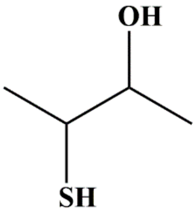

2-mercapto-3-butanol

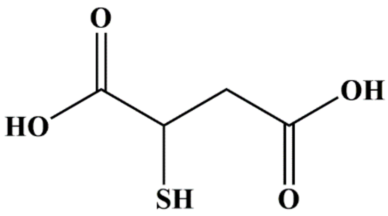

2-mercaptosuccinic acid

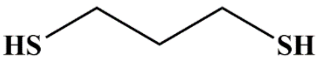

1,3-dimercaptopropane

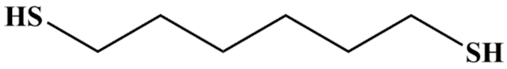

1,6-dimercaptohexane

Figure S2. Structures of 10 sulfhydryl compounds

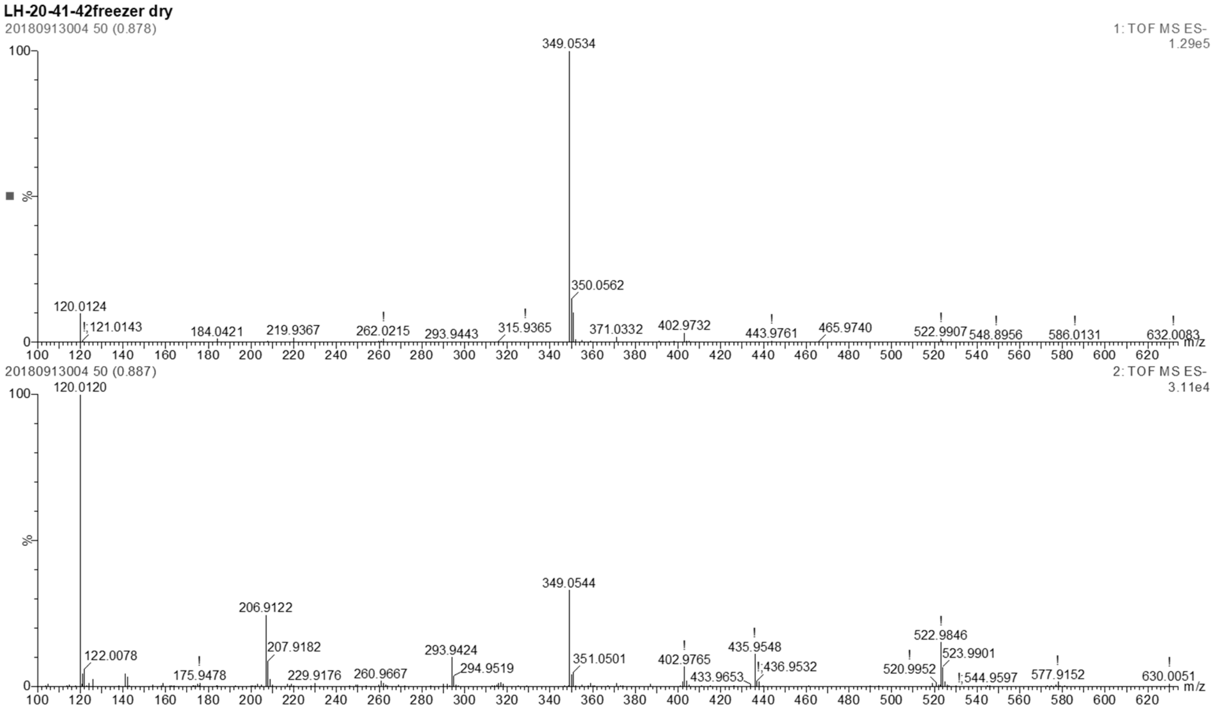

Figure S3. Mass Spectrum of DTH

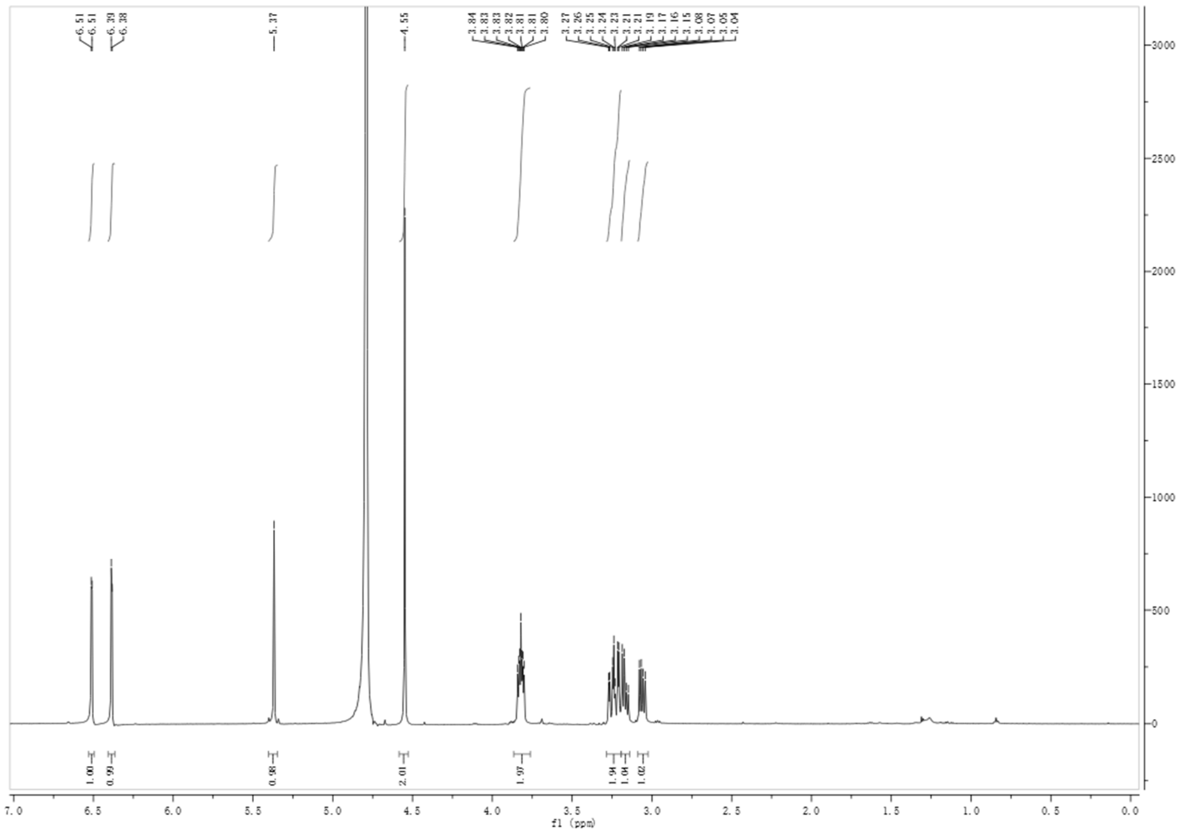

Figure S4. ^{1}H-NMR Spectrum of DTH

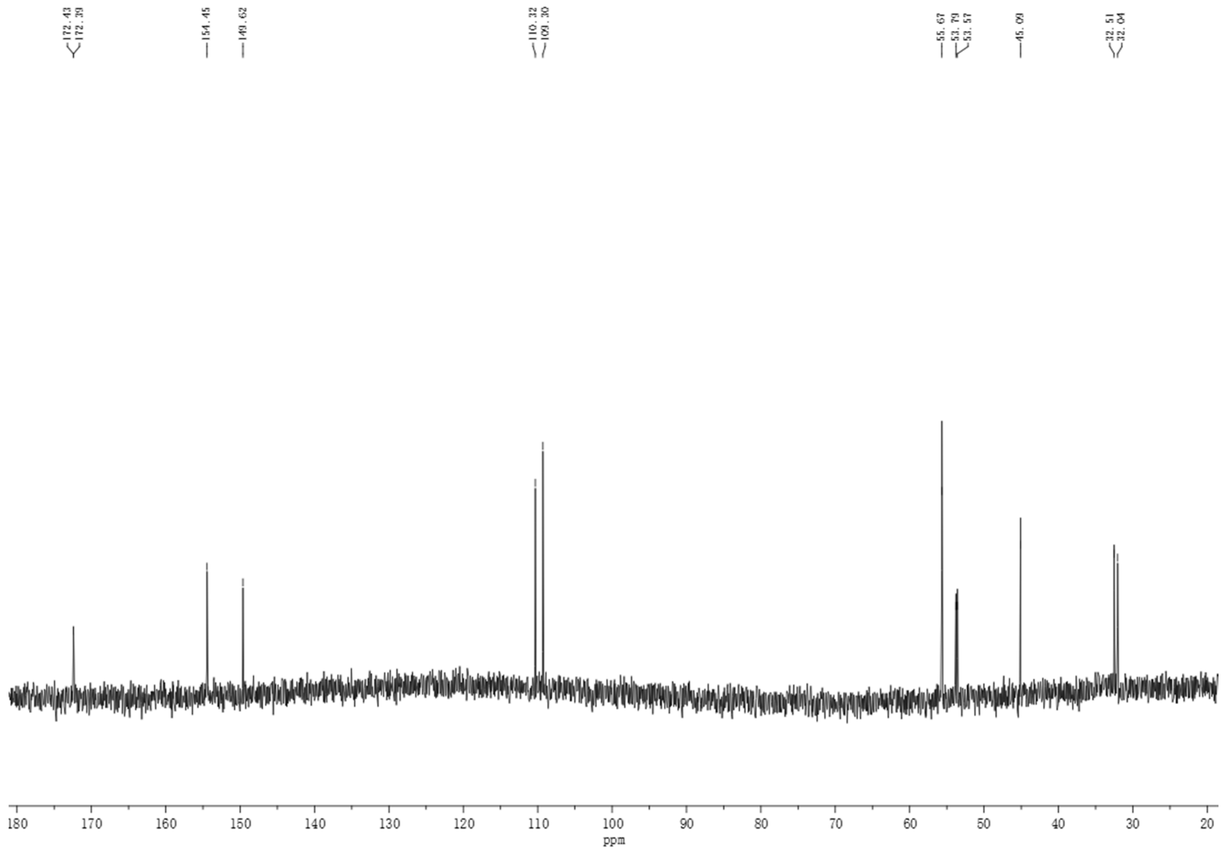

Figure S5. ^{13}C-NMR Spectrum of DTH
